# Protective Effects of Kaempferol against Myocardial Ischemia/Reperfusion Injury in Isolated Rat Heart via Antioxidant Activity and Inhibition of Glycogen Synthase Kinase-3*β*

**DOI:** 10.1155/2015/481405

**Published:** 2015-07-22

**Authors:** Mingjie Zhou, Huanhuan Ren, Jichun Han, Wenjuan Wang, Qiusheng Zheng, Dong Wang

**Affiliations:** ^1^Shandong Provincial Qianfoshan Hospital, Jinan 250014, China; ^2^Weifang Medical University, Weifang 261031, China; ^3^Binzhou Medical University, Yantai 264003, China; ^4^Pharmacy School, Shihezi University, Shihezi 832002, China

## Abstract

*Objective*. This study aimed to evaluate the protective effect of kaempferol against myocardial ischemia/reperfusion (I/R) injury in rats. *Method*. Left ventricular developed pressure (LVDP) and its maximum up/down rate (±*dp*/*dt*_max_) were recorded as myocardial function. Infarct size was detected with 2,3,5-triphenyltetrazolium chloride staining. Cardiomyocyte apoptosis was determined using terminal deoxynucleotidyl nick-end labeling (TUNEL). The levels of creatine kinase (CK), lactate dehydrogenase (LDH), malondialdehyde (MDA), superoxide dismutase (SOD), glutathione/glutathione disulfide (GSH/GSSG) ratio, and tumor necrosis factor-alpha (TNF-*α*) were determined using enzyme linked immunosorbent assay (ELISA). Moreover, total glycogen synthase kinase-3*β* (GSK-3*β*), phospho-GSK-3*β* (P-GSK-3*β*), precaspase-3, cleaved caspase-3, and cytoplasm cytochrome C were assayed using Western blot analysis. *Results*. Pretreatment with kaempferol significantly improved the recovery of LVDP and ±*dp*/*dt*_max_, as well as increased the levels of SOD and P-GSK-3*β* and GSH/GSSG ratio. However, the pretreatment reduced myocardial infarct size and TUNEL-positive cell rate, as well as decreased the levels of cleaved caspase-3, cytoplasm cytochrome C, CK, LDH, MDA, and TNF-*α*. *Conclusion*. These results suggested that kaempferol provides cardioprotection via antioxidant activity and inhibition of GSK-3*β* activity in rats with I/R.

## 1. Introduction

Nowadays, cardiovascular diseases are responsible for the majority of elderly mortality [1]; the most important presentation of cardiovascular disease is ischemia. A long period of ischemia leads to myocardial injury. Theoretically, restoring blood supply to the ischemic myocardium can reduce myocardial injury. However, reperfusion can aggravate myocardial damage through ischemia-reperfusion (I/R) injury [2]. Excessive reactive oxygen species (ROS), calcium overload, inflammatory reaction, and other factors can lead to cellular necrosis, apoptosis, and organ dysfunction in severe cases [3]. Prevention of I/R is important to alleviate ischemic heart disease [4].

Network pharmacology is a method for treating polygenic diseases based on the target and drug perspectives. Mapping the polypharmacology network onto the human disease-gene network can reveal important drug targets that could demonstrate the mechanism of botanical drugs in treating different diseases. Our previous studies identified the potential targets of kaempferol in cardiovascular diseases [5]. Thirteen potential targets were identified and annotated to have significant relationships with the pharmacologic effects of kaempferol. Among these targets, the main protein involved in I/R injury is glycogen synthase kinase-3 beta (GSK-3*β*). GSK-3*β* is a serine/threonine kinase that participates in various cell activities through phosphorylation of the substrate protein [6]. GSK-3*β* is important in glycogen metabolism, as well as in cell proliferation, growth, and death [7, 8]. GSK-3*β* has received increasing attention because of its involvement in some common and serious diseases, such as neurological disease, cancer, and I/R injury. In the cardiovascular system, GSK-3*β* has major roles in glucose metabolism, cardiomyocyte hypertrophy [9], and cell death [10]. Many studies have shown that GSK-3*β* inhibition during I/R is an important mechanism of myocardial adaptation; cardioprotective agents use the inhibition of GSK-3*β* (phosphorylation) as the common downstream target [11], and protection is related to the mitochondrial permeability transition pore (mPTP) [12].

Epidemiological studies have demonstrated that some flavonoids may affect treatment for several diseases [13]. A research on the plants used in traditional medicines revealed that flavonoids are their common bioactive constituents [14]. The flavonoid kaempferol, a yellow compound with low molecular weight (MW: 286.2 g/mol), is commonly found in plant-derived foods and in plants used in traditional medicines. Numerous preclinical studies have shown that kaempferol has a wide range of pharmacological activities, including antioxidant [15], anti-inflammatory [16], and anticancer activities [17].

Therefore, we aimed to evaluate the cardioprotective effects of kaempferol and the mechanisms underlying such effects in the present study.

## 2. Methods

### 2.1. Animals and Reagents

All procedures were performed in accordance with the National Institutes of Health Guideline on the Use of Laboratory Animals and were approved by the Shihezi University Committee on Animal Care. Adult male Sprague-Dawley rats (250–280 g) were obtained from the Xinjiang Medicine University Medical Laboratory Animal Center (SDXK 2011-004) and housed in a room with temperature of 22–25°C, relative humidity of 50–60%, and a 12-h light/12-h dark cycle.

Kaempferol (purity ≥ 98%) was purchased from Shanghai Lichen Biotechnology Co., Ltd. (Shanghai, China). Antibodies against total GSK-3*β*, as well as P-GSK-3*β* (Ser9), caspase-3, and cytoplasm cytochrome C, were obtained from Cell Signaling Technology (1 : 10000, Beverly, MA, USA). Terminal deoxynucleotidyl nick-end labeling (TUNEL) assay was conducted using in situ cell death detection kit (POD, Roche, Germany). All other reagents were of standard biochemical quality and were obtained from commercial suppliers.

### 2.2. Establishment of Animal Model of Myocardial I/R Injury

The rats were randomly divided into four groups as follows: control group, I/R group, kaempferol group, and TDZD-8 (4-benzyl-2-methyl-1,2,4-thiadiazolidine-3,5-dione) group. Hearts from control group were perfused for 120 min stabilization. Hearts from I/R group were stabilized for 20 min and then subjected to 15 min of global ischemia and 85 min of reperfusion. Hearts in kaempferol group were treated with K-H buffer containing kaempferol (15 mmol/L) for 10 min after being stabilized and then subjected to global ischemia for 15 min and reperfusion for 85 min. Hearts in TDZD-8 group were treated with K-H buffer containing TDZD-8 (0.01 mmol/L) for 10 min after being stabilized and then subjected to global ischemia for 15 min and reperfusion for 85 min.

### 2.3. Heart Isolation and Perfusion

Rats were anesthetized with an intraperitoneal injection of 60 mmol/L chloral hydrate (0.35 g/kg) and provided with 250 U/kg heparin through sublingual venous injection to prevent coagulation. The hearts were quickly removed and mounted on a Langendorff apparatus via the aorta for retrograde perfusion with Krebs-Henseleit (K-H) buffer at constant pressure (10 KPa) and constant temperature (37°C). The composition of K-H buffer (in mmol/L) was as follows: NaCl, 118; KCl, 4.7; MgSO_4_, 1.2; CaCl_2_, 2.5; KH_2_PO_4_, 1.2; NaHCO_3_, 25; glucose, 11. The buffer was saturated with 95% O_2_/5% CO_2_ (pH 7.4) [18]. The left atrial appendage was cut. A latex balloon filled with water was inserted into the left ventricle through the left atrial appendage. Finally, hemodynamic parameters, LVDP (LVSP is left ventricular systolic pressure; LVEDP is left ventricular end-diastolic pressure; LVDP = LVSP − LVEDP), ±*dp*/*dt*_max_ (reflecting the important indicators of left ventricular systolic function and diastolic function), CF, and HR, were displayed on the recorder screen.

### 2.4. Evaluation of Myocardial Infarct Size (IS)

After reperfusion was concluded, the heart was frozen at −20°C and cut into five slices along the transverse direction. Each piece was 2 mm thick. The slices were incubated in 1% triphenyltetrazolium chloride (TTC) at 37°C for 20 min. The heart slices were imaged using a digital camera. Area at risk (AAR) and IS were digitally measured using Image Pro Plus software [19]. Myocardial IS was expressed as the ratio between IS and AAR.

### 2.5. Assay of Cellular Injury

To determine the activities of lactate dehydrogenase (LDH) and creatinine kinase (CK), the samples were collected from the coronary effluent before ischemia and after 85 min of reperfusion. The levels of LDH and CK in the effluent were detected spectrophotometrically using their corresponding cytotoxicity detection kits (Nanjing Jiancheng Biological Product, Nanjing, China).

### 2.6. Assay of Oxidative Stress

After perfusion, we obtained the same part of the ventricular apical. Subsequently, the tissue was homogenized in appropriate buffer and centrifuged, after which the supernatant was removed. The levels of superoxide dismutase (SOD) and malondialdehyde (MDA) and glutathione (GSH)/GSH disulfide (GSSG) ratio were analyzed spectrophotometrically using their corresponding assay kits (Nanjing Jiancheng Biological Product, Nanjing, China).

### 2.7. Assay of Inflammation

Tumor necrosis factor alpha (TNF-*α*) was analyzed spectrophotometrically according to the instructions in the TNF-*α* ELISA kit (Tsz Biosciences, Greater Boston, USA).

### 2.8. TUNEL Assay

TUNEL assay was carried out according to the manufacturer's instructions. After deparaffinization and rehydration, the sections were treated with 10 mmol/L protease K for 15 min. The slides were immersed in TUNEL reaction mixture for 60 min at 37°C in a humidified atmosphere in the dark. A converter POD was used to incubate the slides for 30 min. The slides were then analyzed using an optical microscope. To evaluate the apoptosis index of the TUNEL-stained heart tissues, we captured 10 random fields per tissue section at 400x magnification. TUNEL index (%) is calculated as the ratio of the number of TUNEL-positive cells divided by the total number of cells [20] (see Figure 4).

### 2.9. Western Blot Analysis

The protein levels of total GSK-3*β*, phospho-GSK-3*β* (P-GSK-3*β*, Ser9), precaspase-3, cleaved caspase-3, and cytoplasm cytochrome C were determined using Western blot analysis. After perfusion by Langendorff apparatus, we cut off the same part in ventricular apical of the rats, then homogenized the cut tissue in appropriate buffer, and centrifuged it. Supernatant was extracted and boiled for 15 min to make protein denaturation. Then the whole-cell protein extracts were separated using 12% SDS-polyacrylamide gel electrophoresis. Proteins were transferred to nylon membranes by electrophoretic transfer system. The membranes were blocked with 5% skimmed milk blocking buffer at room temperature for 1 h and then incubated with primary antibodies overnight (18 h) at 4°C. After being washed with TBST buffer, the corresponding secondary antibodies were used to identify primary antibody binding. In the end, the blots were visualized with ECL-plus reagent.

### 2.10. Statistical Analysis

Results were expressed as mean ± S.D. and analyzed using one-way analysis of variance. The values with *P* < 0.05 were considered statistically significant. All statistical tests were performed with GraphPad Prism software version 5.0 (GraphPad Software, San Diego, CA).

## 3. Results

### 3.1. Kaempferol Improved the Recovery of I/R-Induced Cardiac Function

As shown in Table 1, the hearts in the I/R group demonstrated significant decrease in the recovery of cardiac function compared with that in the control group. Moreover, the hearts in the kaempferol and TDZD-8 treatment groups showed higher recovery of cardiac function than that in the I/R group (*P* < 0.05). Hemodynamic data confirmed that kaempferol improved the recovery of the cardiac systolic and diastolic function of the rats after I/R.

### 3.2. Kaempferol Reduced Myocardial IS Post-I/R

**Figure 1:**
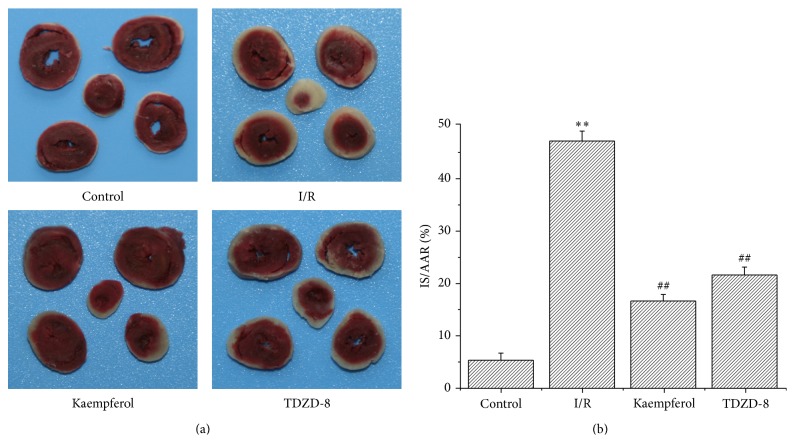
Kaempferol reduces the I/R-induced IS. (a) Images of myocardial tissue sections after TTC staining. (b) The ratio between IS and AAR, in which AAR is the area at risk and IS is the infarct size; values are presented as means with their standard deviations ($\overset{-}{x}\pm s$, *n* = 8); ^*∗∗*^ *P* < 0.01, compared with the control group; ^##^ *P* < 0.01, compared with the I/R group.

The representative slices of the hearts are shown in Figure 1(a). The ratio of IS and AAR in the control group was 5.31% ± 1.34%, which was significantly different from that of the I/R group (46.73% ± 1.88%) (*P* < 0.01; Figure 1(b)). The ratio remarkably decreased in the kaempferol (16.49% ± 1.23%) and TDZD-8 groups (21.42% ± 1.48%) compared with that in the I/R group (*P* < 0.01; Figure 1(b)).

### 3.3. Kaempferol Attenuated the I/R-Induced Enzyme Release in Rat Heart

As shown in Table 2, the levels of perfusate CK and LDH in the I/R group (56.74 ± 2.97 and 60.54 ± 2.35, resp.) significantly increased compared with that in the control group (*P* < 0.01); however, the levels of CK and LDH in the kaempferol (32.30 ± 2.48 and 36.63 ± 1.83, resp.) and TDZD-8 groups (36.73 ± 2.54 and 39.67 ± 1.64, resp.) (*P* < 0.01) were significantly decreased compared with the I/R group.

### 3.4. Kaempferol Improved the Oxidative Stress State Induced by I/R

**Figure 2:**
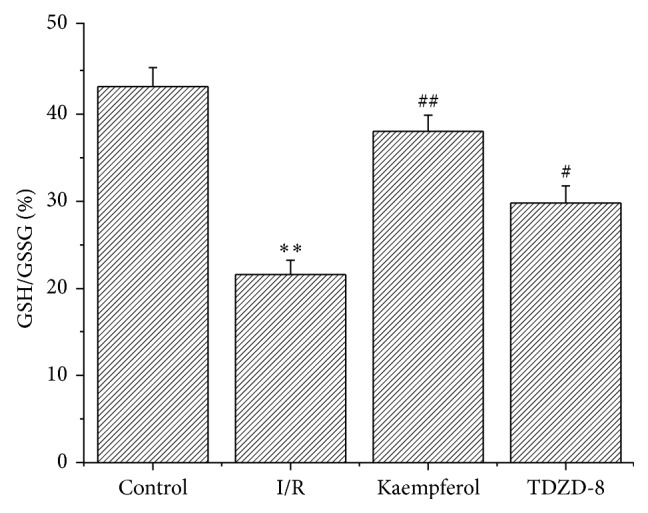
Kaempferol increases the ratio of GSH/GSSG. Values are presented as means with their standard deviation ($\overset{-}{x}\pm s$, *n* = 8); ^*∗∗*^ *P* < 0.01, compared with control group; ^##^ *P* < 0.01 and ^*#*^ *P* < 0.05, compared with the I/R group.

To identify the possible mechanisms of kaempferol involving antioxidants on cardioprotection, we evaluated SOD activity, MDA level, and GSH/GSSG ratio in the myocardial tissues. As shown in Table 3, and Figure 2, the SOD activity and the ratio of GSH/GSSG in the kaempferol or TDZD-8 groups significantly increased, compared with those of the control group, whereas the MDA level significantly decreased in the kaempferol or TDZD-8 groups.

### 3.5. Kaempferol Reduced the Inflammatory Response

**Figure 3:**
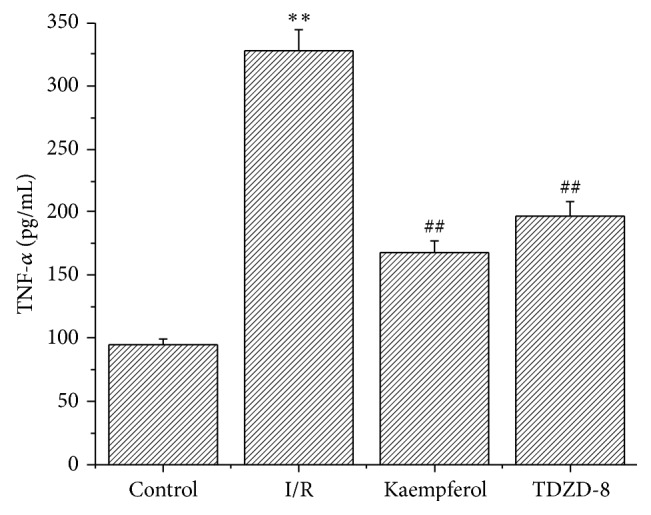
Kaempferol reduces inflammatory response. Values are presented as means with their standard deviations ($\overset{-}{x}\pm s$, *n* = 8); ^*∗∗*^ *P* < 0.01, compared with the control group; ^##^ *P* < 0.01, compared with the I/R group.

**Figure 4:**
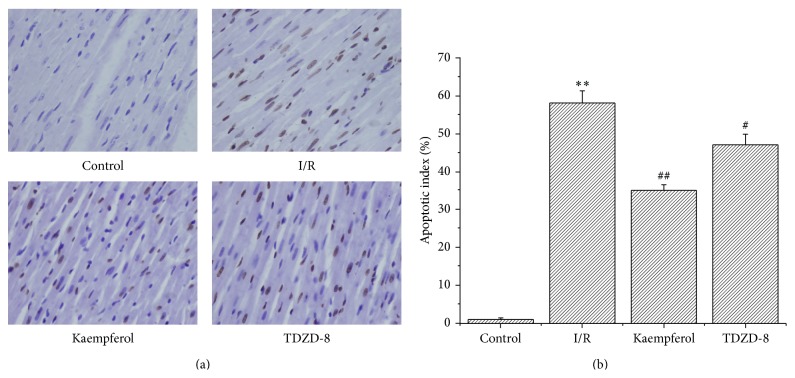
Kaempferol prevents myocardial cell apoptosis in I/R. (a) Cell apoptosis was analyzed using TUNEL staining. Magnification: 400x. (b) Apoptosis cells/total cells (%). Values are presented as means with their standard deviations ($\overset{-}{x}\pm s$, *n* = 8); ^*∗∗*^ *P* < 0.01, compared with the control group; ^##^ *P* < 0.01 and ^#^ *P* < 0.05, compared with the I/R group.

As shown in Figure 3, the level of TNF-*α* increased from 95 ± 4.5 pg/mL in the control group to 328 ± 16.7 pg/mL in the I/R group. The levels of TNF-*α* in the kaempferol (168 ± 9.3 pg/mL) and TDZD-8 groups (197 ± 11.4 pg/mL) significantly decreased compared with that in the I/R group (*P* < 0.01).

### 3.6. Kaempferol Weakened the Cardiomyocyte Apoptosis Induced by I/R

TUNEL staining did not show a significant apoptotic phenomenon in the control group, whereas the number of apoptotic cells evidently increased in the I/R group (58 ± 3.21). Apoptotic cells significantly decreased in the kaempferol (35 ± 1.48) and TDZD-8 groups (47 ± 2.79) compared with those in the I/R group (*P* < 0.01).

### 3.7. Effects of Kaempferol on GSK-3*β* Phosphorylation, Cytochrome C Release, and Caspase-3 Activity

**Figure 5:**
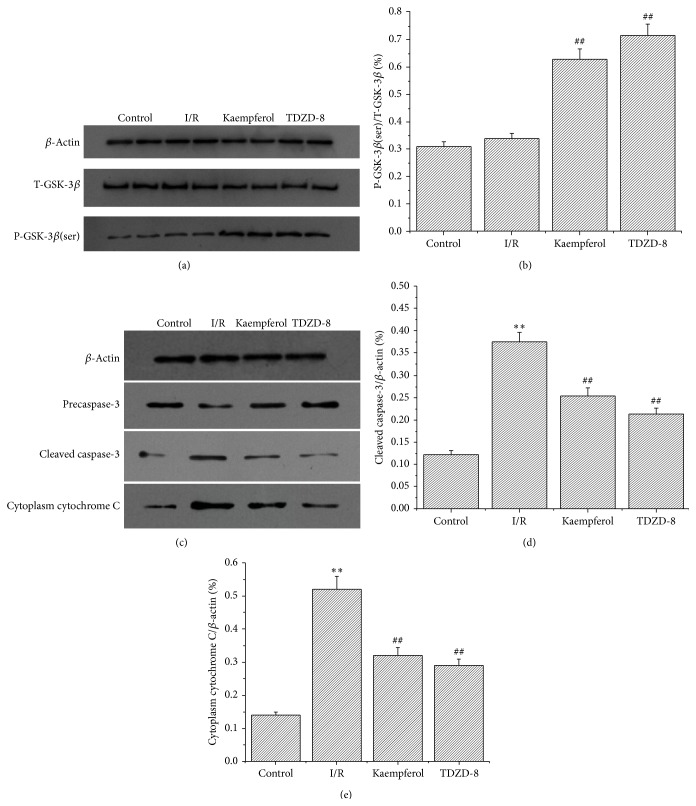
Kaempferol increases the phosphorylation of GSK-3*β* and reduces the release of cytochrome C and the dissociation of caspase-3. (a) Representative Western blots for T-GSK-3*β* and P-GSK-3*β* (Ser9). Lanes 1 and 2 are the control group, lanes 3 and 4 are the I/R group, lanes 5 and 6 are the kaempferol group, and lanes 7 and 8 are the TDZD-8 group. (b) Grey value analysis demonstrates that kaempferol increases the ratio of P-GSK-3*β* (ser)/T-GSK-3*β*. (c) Representative Western blot for cleaved caspase-3 and cytoplasm cytochrome C. Lane 1 is the control group, lane 2 is the I/R group, lane 3 is the kaempferol group, and lane 4 is the TDZD-8 group. (d) Grey value analysis demonstrates that kaempferol reduces the ratio of cleaved caspase-3/*β*-actin. (e) Grey value analysis demonstrates that kaempferol reduces the ratio of cytoplasm cytochrome C/*β*-actin. Values are presented as means with their standard deviations ($\overset{-}{x}\pm s$, *n* = 8); ^*∗∗*^ *P* < 0.01, compared with the control group; ^##^ *P* < 0.01, compared with the I/R group.

As shown in Figure 5, the level of GSK-3*β* phosphorylation in the kaempferol (0.65 ± 0.039) and TDZD-8 groups (0.74 ± 0.043) significantly increased compared with those in the control (0.32 ± 0.018) and I/R groups (0.35 ± 0.02) (*P* < 0.01). However, no evident difference was observed between the control and I/R groups. The release of cytochrome C and the dissociation of caspase-3 in the I/R groups (0.52 ± 0.039 and 0.37 ± 0.021, resp.) were significantly increased compared with those in the control groups (*P* < 0.01), whereas the kaempferol (0.32 ± 0.024 and 0.25 ± 0.018, resp.) and TDZD-8 groups (0.21 ± 0.013 and 0.29 ± 0.019, resp.) were significantly decreased compared with those in the I/R groups (*P* < 0.01).

## 4. Discussion

In this study, we investigated the effects of kaempferol on cardiac function, myocardial IS, cardiomyocyte apoptosis, inflammation factor, and myocardial enzyme in the isolated rat heart model of I/R. We provided evidence that kaempferol improves the recovery of cardiac function, reduces intracellular oxidation status and myocardial IS, and inhibits myocardial apoptosis induced by I/R. Finally, we demonstrated that the phosphorylation of GSK-3*β* is involved in the cardioprotection of kaempferol.

Reactive oxygen species (ROS) induced injury plays an important role in the development of I/R in various organs [21]. Few free radicals are present under physiological conditions during ischemia; thus, the absorbance of oxygen radical decreases [22]. During recovery, the blood supply of the tissues triggers the “explosion” of oxygen free radicals; hence, the accumulated ROS attack the cells and cause damage [23]. ROS causes damage to the cell membranes in rat heart, subsequently causes cell membrane lipid peroxidation and structural failure, and results in leakage of myocardial enzyme. Inhibiting the ROS generation or the antagonist of reactive oxygen toxicity is important to alleviate myocardial reperfusion injury [24]. In the present experiment, SOD activity and GSH/GSSG ratio significantly increased, whereas MDA level significantly decreased in the kaempferol group. Therefore, we speculated that kaempferol has good antioxidant effect that functions as myocardial protection.

During I/R, oxidative stress activates NF-*κ*B because cardiac stress rapidly responds to the expressed genes. NF-*κ*B stimulates the cardiac cells and macrophages and produces large amounts of TNF-*α*; thus, the myocardial cells after I/R are the sites for TNF-*α* synthesis and the target organs of TNF-*α*. Studies have shown that TNF-*α* dose dependently decreases myocardial contractility, whereas the application of anti-TNF-*α* reduces I/R injury and confers protection for the ischemic myocardium [25]. The TNF-*α* bioactivity in the heart increases from the early stage of I/R; such increase has been speculated to partially contribute to the increased area of myocardial infarction [26]. The ROS generated within cells can induce apoptosis through multiple pathways. Cardiomyocytes, which are abundant in the mitochondria, are the major endogenous source and are the susceptible target of ROS damage [27]; the abundant ROS within the mitochondria can decrease its selective ion permeability or change its membrane permeability, which changes the mitochondrial membrane and thereby induces mitochondrial permeability transition pore (MPTP) opening during reperfusion [28]. The mitochondria release cytochrome C, which activates the aspartate-specific cysteine proteases (caspases) and induces apoptosis [29].

Cytochrome C is located within the intermembrane space under normal conditions; the released cytochrome C binds to the C-terminal domain of the apoptotic protease activating factor-1 (Apaf-1) and changes its conformation [30]. The activated Apaf-1/cytochrome C complex promotes caspase activation [31]. Several studies in the literature showed that GSK-3*β* inhibition might delay or suppress mPTP opening and inhibit the release of cytochrome C [32, 33]. TDZD-8 is a GSK-3*β* inhibitor with significant myocardial protection effect via inhibition of inflammation and apoptosis [34]. We used TDZD-8 as a positive control agent to demonstrate the mechanisms of the cardioprotective effects of kaempferol. Our research showed that kaempferol or TDZD-8 can increase the level of GSK-3*β* phosphorylation and reduce the release of cytochrome C compared with those in the control and I/R groups. We inferred that kaempferol can function similarly to that of TDZD-8 on GSK-3*β* phosphorylation. Thus, we speculated that kaempferol decreases the apoptosis induced by I/R injury via GSK-3*β* inhibition.

## Figures and Tables

**Table 1:** Effect of kaempferol on cardiac function in the rats subjected to I/R ($\bar{x}\pm s$, %, *n* = 8); ^*∗∗*^ *P* < 0.01, compared with the control group; ^##^ *P* < 0.01, compared with the I/R group.

Reperfusion	Control	I/R	Kaempferol	TDZD-8
LVDP (mmHg)	87.98 ± 3.98	52.43 ± 2.62^*∗∗*^	69.47 ± 2.26^##^	64.53 ± 2.47^##^
+LV*dp/dt*_max_ (mmHg·s^−1^)	89.37 ± 3.73	45.86 ± 2.53^*∗∗*^	63.27 ± 2.76^##^	60.58 ± 2.96^##^
−LV*dp/dt*_max_ (mmHg·s^−1^)	86.76 ± 3.56	42.16 ± 2.38^*∗∗*^	61.54 ± 3.14^##^	60.79 ± 3.35^##^
CF (mL·min^−1^)	82.19 ± 3.63	51.46 ± 2.36^*∗∗*^	67.21 ± 4.06^##^	64.59 ± 3.97^##^
HR (beats·min^−1^)	90.51 ± 4.58	70.68 ± 4.87^*∗∗*^	83.92 ± 3.99^##^	78.68 ± 4.13^##^

**Table 2:** Effect of kaempferol on the levels of CK and LDH in the coronary effluent before ischemia and after 85 min of reperfusion ($\bar{x}\pm s$, *n* = 8); ^*∗∗*^ *P* < 0.01, compared with the control group; ^##^ *P* < 0.01, compared with the I/R group.

Groups	Before ischemia	Reperfusion
CK (U/L)		
Control	16.73 ± 1.46	24.81 ± 1.31
I/R	15.76 ± 1.21	56.74 ± 2.97^*∗∗*^
Kaempferol	16.72 ± 1.23	32.30 ± 2.48^##^
TDZD-8	16.45 ± 1.26	36.73 ± 2.54^##^
LDH (U/L)		
Control	14.47 ± 1.36	19.51 ± 1.35
I/R	14.58 ± 1.38	60.54 ± 2.35^*∗∗*^
Kaempferol	15.35 ± 0.96	36.63 ± 1.83^##^
TDZD-8	15.49 ± 1.12	39.67 ± 1.64^##^

**Table 3:** Effects of kaempferol on SOD activity and MDA level. Values are presented as means with their standard deviations ($\bar{x}\pm s$, *n* = 8); ^*∗∗*^ *P* < 0.01, compared with the control group; ^##^ *P* < 0.01 and ^#^ *P* < 0.05, compared with the I/R group.

Group	Dosage	Reperfusion	
		SOD (U/mgPr)	MDA (nmol/mgPr)
Control	—	10.48 ± 0.79	210.43 ± 12.56
I/R	—	3.35 ± 0.41^*∗∗*^	489.71 ± 30.25^*∗∗*^
Kaempferol	15 mmol/L	8.15 ± 0.57^##^	285.76 ± 19.04^##^
TDZD-8	0.01 mmol/L	5.43 ± 0.62^#^	370.24 ± 22.50^##^
